# Shaping and Structuring of Polymer Gels

**DOI:** 10.3390/gels10020134

**Published:** 2024-02-07

**Authors:** Toshiaki Dobashi

**Affiliations:** Gunma University, Kiryu 376-8515, Japan; dobashi@gunma-u.ac.jp

Most industrial gels are prepared as apparently isotropic and homogeneous materials through a preparation process encompassing alterations in temperature, application of isotropic mechanical stress, exposure to high-energy electromagnetic waves, and mixing with cross-linkers (gelators). On the other hand, gels featuring spatially resolved structures and mechanical properties could help functionalize materials with superior performance across diverse fields. For example, the gel-based micro/nano-patterning of electrical pathways within gel matrices facilitates the fabrication of sophisticated soft devices [1]. Non-uniform internal stress in gels is frequently a pivotal factor in the development of self-shaping and actuating materials and is particularly relevant in applications such as biosensing, micro-robotics, and optics [2]. In tissue engineering, the creation of anisotropic organs and tissues, such as nerve fibers, relies on degradable gel scaffolds [3]. The distinctive shapes and structures of these gels are achieved through various methods, including the diffusion of gelators, 3D printing, photopatterning, and surface-mediated approaches. These methodologies are likewise prevalent in the shaping and structuring of supramolecular gels [4]. This Special Issue is dedicated to showcasing recent research and advancements in the shaping and structuring of polymer gels. The collection comprises ten articles elucidating the fabrication of gels with well-defined shapes and spatially controlled structures, the measurement and analysis of the chemical and rheological properties of anisotropic gels, and theoretical and experimental insights into anisotropic gelation dynamics. Importantly, these investigations explore the practical applications of such advancements in biomedical and food technology.

Collagen, a significant extracellular matrix constituent, has been widely studied for its utility in tissue engineering applications owing to its inherent biocompatibility and regulatory roles in modulating cellular morphology and functions. Different forms of collagen have been prepared to align with the specific requisites of diverse applications. Furusawa et al. endeavored to formulate a methodology to generate gels that emulate the structural attributes of skeletal muscle. This was achieved through the cultivation of chicken embryonic muscle cells in genipin-crosslinked multi-channel collagen gels (MCCG) [5]. The resultant myotubes exhibited distinct anisotropic elasticity and relaxation strengths coupled with intriguing contractile responses to periodic electrostimulations within a frequency window of 0.5–2.0 Hz (Contribution 1). In a complementary scholarly contribution (Contribution 2), Ishibashi et al. harnessed their one-pot method [6] to fabricate collagen tube gels, which yielded hollow collagen gels as mimetic models of blood vessels. Notably, their demonstration showed the feasibility of UV-treated collagen pre-gel solutions for preparing collagen tubular gels endowed with sufficient hollowness.

Porosity is one of microstructural characteristics of gels for drug delivery systems. Because of their intricate porous network architecture, hydrogels can be extensively used in medicine, where they serve as carriers for precisely controlled drug delivery in which drugs are loaded into the porous framework of hydrogels, allowing for the gradual diffusion of small polymer or oligomer molecules throughout the gel network, thereby effectuating controlled release [7]. Micropores formed in gels using micro/nano-bubbles as templates function as channels that facilitate the retention and efflux of water molecules. Kuroki et al. demonstrated that employing bubble water as a solvent in the fabrication of the light-induced volume phase transition of gels accelerates the transition rate by approximately 100-fold compared with using gels without bubble water. This innovative approach significantly augments hydrogel response rates without relying on additives (Contribution 3). The microstructure of hydrogels often exhibits notable divergence that is contingent upon the polymer source. Zhao et al. studied the physicochemical properties and microstructure of hybrid hydrogels prepared using sodium alginate (SA) and chondroitin sulfate (CS) sourced from two animal origins. Their findings elucidate that SA-based hybrid hydrogels incorporating chicken-derived CS have a more compact porous microstructure and superior interfacial compatibility in comparison with those incorporating bovine-derived CS (Contribution 4).

The bacterial fermentation of dairy products such as fresh cheese and yogurt is mimicked by glucono-δ-lactone (GDL)-induced acid gel. The elucidation of mesoscopic information is imperative for the enhancement of textural and physical attributes in acidified milk products. Recent advancements in imaging microscopy have made it a powerful tool in studying mesoscopic dynamics, as demonstrated in the investigation of rennet-induced gelation [8]. Sekiguchi et al. studied the gelation kinetics of acidified milk using several advanced image analysis techniques, namely particle image velocimetry, differential variance analysis, and differential dynamic microscopy, utilizing fat globules as discerning probes. They successfully revealed that, in the very early process of acidification, microscopic viscosity develops in two steps (Contribution 5).

Supramolecular gels are one of essential materials in modern technology with applications in substance separation within chemical processes, technology for environmental cleanup, biotechnology, drug development, and regenerative medicine [9]. Broadband dielectric spectroscopy is a potent investigative tool to elucidate the formation of hydrogels’ molecular structures. Shimizu et al. applied this technique to study the formation of fibrous supramolecular gels using a low-molecular-weight gelator. Their results suggested a structure formation of rod micelles appearing as precursors before cross-linking into the three-dimensional network of the supramolecular gels and substantiated the validity of their effective relaxation parameter analysis for understanding details of the gelation mechanism (Contribution 6). While dielectric spectroscopy captures relaxation phenomena reflecting the dynamic aspects and time correlations of physical quantities, spatial structural information remains conspicuously absent. In Contribution 7, Yagihara et al. employed the ergodic hypothesis in relaxation theory. Their success lay in the utilization of *τ–β* diagrams to analyze spatial information, where *τ* and *β* are the relaxation time and distribution parameter, respectively [10]. Particularly noteworthy are their revelations concerning the spatial distribution of water molecules within the diverse systems encompassing polymer gels, supramolecular gels composed of surfactant micelles, and cement gels.

Gelation occasionally exhibits concomitantly with pattern formation. Haraguchi et al. discerned unique concentric and radial macroscopic spatial patterns during the deposition of a calcium nitrate solution onto the center of a sodium alginate solution in a Petri dish. The concentric patterns had the same spatial rules as the well-established Liesegang pattern [11]. The radial pattern manifested as surface cracks in the hydrogel. The required situation and the tendency toward equal spacing resembled the horizontal banding formed in the drying process to monitor crack propagation. These patterns were expected due to the competition between gelation and phase separation in the aqueous solution of a polysaccharide–metal ion system, driven by the diffusion of metal ions into the polysaccharide solution (Contribution 8).

When gelation initiates at a point or a surface in a solution, a distinct gel region evolves, and the extent of gelation is quantified by the resultant gel volume. The theoretical framework governing such gelation markedly diverges from that for isotropic gelation. Yamamoto et al. directed their focus to the emergence of a discernible sol–gel interface during the gelation process. They expounded the motion of this interface, denoted as the moving boundary (MB), rooted in the principles of non-equilibrium thermodynamics. The main conclusion of the theory is that the dynamics are expressed by scaled equations that are categorized into several types depending on the gelation mechanism and geometrical condition [12]. In Contribution 9, Kakinoki et al. studied a novel genre of gelation induced by the diffusion of enzyme molecules recurrently employed for cross-linking. Their investigation revealed that upon the application of an enzyme solution onto a physical isotropic gelatin gel, an ensuing isotropic chemical gelation transpires, succeeded by gel polymer orientation following a significant lag time. The gelation dynamics of the system were elucidated through a combination of diffusion-limited gelation followed by the free-energy-limited orientation of polymer molecules. Contribution 10, authored by Yamamoto, extended the theoretical paradigm to encompass scenarios wherein the state of polymer solutions undergoes transformation due to the influx of gelators, rendering the sol phase metastable and the gel phase stable. Notably, in the dynamics of gel growth, a deviation from scaling law was observed in the early stage, only to conform in the late stage. The article dissects the crossover phenomenon within the context of scaling, shedding light on the rate-limiting processes inherent in liquid–liquid contact-induced gelation.

In conclusion, the scholarly domain of Shaping and Structuring of Polymer Gels is growing remarkably with a continuous influx of novel methodologies for the preparation and characterization of gels. This extends particularly to the mesoscale spatial order and a broad spectrum of temporal scales, with a discerning focus on the theoretical underpinnings of anisotropic gelation. These advancements underscore the potential for diverse applications across various scientific and technological realms. The thematic fabric of nearly all contributions is interwoven with biomaterials, aligning their objectives with biological and biomedical applications. It is notable that the anisotropy inherent in gels mirrors the natural intricacies of certain physiological components in our bodies. The exploration of the role of diffusion, based on “gradients” focused in a pioneering book by Child [13], as posited by Crick, traces back to 1970 [14]. Contemporary insights into anisotropic morphogenesis in developmental biology have been comprehensively summarized in review articles [15,16]. While the detailed mechanisms governing biosynthesis remain complex, there is anticipation that certain facets of this process can be artificially recreated.
